# “The Cancer is My Life”: patient and caregiver perceptions of the time toxicity of palliative systemic cancer treatments for advanced gastrointestinal cancers

**DOI:** 10.1007/s00520-025-09621-4

**Published:** 2025-06-10

**Authors:** Samuel X. Stevens, Ella El-Katateny, Richard De Abreu Lourenço, Christopher M. Booth, Joanne Shaw, Janette L. Vardy

**Affiliations:** 1 Sydney School of Medicine, Faculty of Medicine and Health, The University of Sydney, Camperdown, Sydney, NSW 2008 Australia; 2 Concord Repatriation General Hospital, Concord West, NSW Australia; 3 School of Psychology, The University of Sydney, Sydney, NSW Australia; 4 Centre for Health Economics Research and Evaluation, University of Technology Sydney, Sydney, NSW Australia; 5 Departments of Oncology and Medicine, Queen’s University, Kingston, ON Canada

**Keywords:** Time toxicity, Advanced cancer, Gastrointestinal cancers, Qualitative, Supportive care

## Abstract

**Purpose:**

Treatment for advanced cancer entails substantial time commitments, which has been labelled the ‘time toxicity’ of treatment, though the perspectives of people affected by cancer are still being established. We aimed to establish patient and caregiver perspectives on the ‘time toxicity’ of palliative systemic treatments.

**Methods:**

Semi-structured qualitative interviews were conducted using an inductive approach. Purposively selected adults with advanced gastrointestinal cancers who had received palliative systemic therapy and caregivers were recruited from one metropolitan and regional site. Interviews were analysed using thematic analysis.

**Results:**

Twenty patients and ten caregivers participated. Eighty percent were Australian-born, 60% were 55–74 years old, 57% had colorectal cancer, 50% were female, and 50% were regionally situated. Five themes emerged: (1) treatment as work, (2) opportunity costs of receiving care, (3) treatment time as an investment, (4) time in treatment decision-making, and (5) tools for managing treatment time. Participants found it burdensome to organise their lives around treatment requirements. Perception of time burdens related to understandings of treatment benefit, experience of downsides, and psychological reactions to illness. Time spent coordinating and recovering from treatment had a substantial impact on participants’ lives outside of contact days. However, participants valued the potential benefits of treatment and described healthcare time as a modifier, rather than a driver, of treatment decision-making.

**Conclusion:**

This qualitative analysis contributes a foundational understanding of perceptions, sources, and impacts of healthcare time burdens in an Australian context. Further research will identify, assess, and address modifiable sources of time burdens in cancer care.

**Supplementary Information:**

The online version contains supplementary material available at 10.1007/s00520-025-09621-4.

## Introduction

Cancer care demands substantial time and organisational resources from patients and their caregivers. These time commitments have been labelled “time toxicity” [8], a component of treatment burden that specifically focuses on the impact of time traded to receive cancer care. The concept of time toxicity has rapidly gained traction with oncology and lay audiences, and a growing number of studies assess patient healthcare contact time [2, 6, 9, 13, 20, 23–25]. These data suggest adults with advanced solid tumours spend between 10 and 33% of their survival time in contact with the healthcare system. When factoring in healthcare days associated with treatment, systemic treatments with marginal survival benefits can result in less time alive and out of hospital than supportive care alone [8, 12]. This information can inform discussions about the value of cancer treatments for individual patients.

However, we lack an understanding of the impact of healthcare contact on patients and their caregivers. Literature characterising the related concepts of logistic and financial toxicity has demonstrated that objective burdens are variably translated into subjective burdens depending on patient circumstances [5, 31] and highlights the impact of time-intensive tasks, such as coordinating and commuting to care, on perceptions of treatment burden. However, similar work has not yet been done in the field of time toxicity. By exploring perspectives, sources, and consequences of time burdens, we can refine our understanding of time toxicity and identify targets for quality improvement.

Time has high intrinsic value for all people with cancer; however, considerations of time toxicity are particularly germane to patients requiring frequent healthcare contact where prognosis is limited. People living with gastrointestinal (GI) cancers are one such group. GI cancers account for one third of cancer-related deaths globally and disproportionately affect socioeconomically disadvantaged populations [4, 17]. Treatment frequently involves multi-drug, multi-day infusional chemotherapy; patients tend to have high symptom burden and often have multidisciplinary care needs. Whilst treatments are relatively homogenous, affected patients come from diverse backgrounds, and experiences with treatment vary [2, 13, 19, 25].

Cancer care in Australia is most commonly delivered in centralised, publicly funded metropolitan cancer centres [17]. There is considerable variation in resourcing for specialised support staff such as nurse navigators; thus, caregivers are often highly involved in managing symptoms, coordinating healthcare, and providing transport to and from ambulatory appointments. Geographic isolation from major hubs is a substantial barrier to accessing oncologic care, with rurality a significant predictor of worse cancer outcomes [22, 27, 29].

Qualitative research is well placed to clarify the assumptions behind time toxicity, understand its consequences for patients and caregivers, and elucidate its role in treatment decision-making. This study aimed to build a foundational understanding of time toxicity by exploring perceptions of healthcare contact time in patients with advanced GI malignancies and their caregivers across metropolitan and regional Australia.

## Materials and methods

Qualitative semi-structured interviews were conducted to explore perspectives of patients and their unpaid caregivers of the time spent in activities relating to cancer treatment. The study was approved by the Sydney Local Health District (Concord Repatriation General Hospital) Human Research Ethics Committee (ETH/00869). The methods and analysis were reported in accordance with the Consolidated Criteria for Reporting Qualitative Research guidelines [30] (Supplementary Appendix 1).

### Study design and recruitment

We recruited adults with advanced GI cancer who had received at least one cycle of palliative-intent systemic treatment, as well as caregivers, from one metropolitan and one regional cancer centre in New South Wales, Australia. Patient participants could have either de novo metastatic or recurrent disease after curative-intent treatment. Multiple GI cancer types were included to capture the diversity of experiences across disease types, treatments, and prognoses. Caregivers were included due to their frequent and often extensive involvement in activities related to treatment. They could participate independent of the person cared for. Regional participants were included to capture the potential impact of rurality on time spent accessing healthcare.

Purposive sampling was used to recruit participants with diverse experiences of treatment. Potential participants were referred to the study by their treating clinician during routine appointments. Written informed consent was provided using Research Electronic Data Capture (REDCap) eConsent [15, 16]. Participants did not receive compensation for participating.

### Data collection

The recruitment period was 5 October 2023 to 17 July 2024. Participants self-reported age bracket, gender, postcode, education, relationship and carer status, diagnosis, and prior treatment. Postcode information was used to populate the socio-economic index for area (SEIFA), a measure of socio-economic advantage, and the modified Monash model (MMM), which is an Australian model describing remoteness from medical services. Caregivers also completed a 12-item Zarit Burden Interview (ZBI-12) [32]. Interviews were conducted either face-to-face or online. Recruitment continued until thematic saturation was achieved.

Interview guides were developed by the research team drawing on clinical experience and existing literature on time and treatment burdens (Supplementary Appendix 2). Questions were designed to be open-ended to encourage rich discussion, exploring the sources, impact, and consequences of healthcare time and communication of healthcare time in treatment decision-making. The interview guide was refined in consultation with a patient advocate and amended iteratively. Interviews were digitally audio-recorded and transcribed verbatim using TRINT (www.trint.com).

### Analysis

De-identified transcripts were independently reviewed by four members of the study team (SS, EE, JS, JV), and an initial coding framework was developed. The framework was independently applied to interviews (SS, EE) and coded using NVivo 14 software (QSR International; Melbourne, Australia). Any disagreements were discussed with the wider team until consensus was reached. Interview data were analysed using thematic analysis tied to a framework approach [3, 7] (Supplementary Appendix 3) using an inductive, interpretivist approach based in grounded theory. Following coding, the study team reviewed the data, grouped codes and nodes, and identified broad themes (Supplementary Appendix 4). Illustrative quotes are referenced with patient/caregiver (P/C), participant number, and regional or metro (R/M) (e.g. P1-M for patient, participant one, metropolitan location). Further illustrative quotes can be found in Table 1.

**Table 1:** Participant demographics

Characteristic	Group	Patients, *n* (%)	Caregivers, *n* (%)	Total, *n* (%)
Gender	Male	12 (60)	3 (30)	15 (50)
	Female	8 (40)	7 (70)	15 (50)
Age bracket	18–34	1 (5)	2 (20)	3 (10)
	35–54	3 (15)	2 (20)	5 (17)
	55–74	13 (65)	5 (50)	18 (60)
	≥ 75	3 (15)	1 (10)	4 (13)
Geographic location	Metropolitan	10 (50)	5 (50)	15 (50)
	Regional	10 (50)	5 (50)	15 (50)
MMM^a^ classification	1	9 (45)	5 (50)	14 (47)
	2	1 (5)	1 (5)	2 (7)
	3	2 (10)	2 (10)	4 (13)
	4	2 (10)	1 (5)	3 (10)
	5	3 (15)	1 (5)	4 (13)
	6	2 (10)	0 (0)	2 (7)
	7	0 (0)	0 (0)	0 (0)
SEIFA quintile^b^	1	5 (25)	1 (10)	6 (20)
	2	6 (30)	2 (20)	8 (27)
	3	5 (25)	3 (30)	8 (27)
	4	3 (15)	1 (10)	4 (13)
	5	4 (20)	3 (30)	7 (23)
Born in Australia	Yes	16 (80)	6 (60)	24 (80)
English spoken at home	Yes	18 (90)	8 (80)	26 (87)
Relationship status	Single	3 (15)	1 (10)	4 (13)
	Married/de facto	14 (70)	9 (90)	23 (77)
	Separated/divorced	3 (15)	0 (0)	3 (10)
	Widowed	1 (5)	0 (0)	1 (3)
Level of education	High school	10 (50)	5 (50)	15 (50)
	Diploma	7 (35)	2 (20)	9 (30)
	Bachelor Degree	1 (5)	2 (20)	3 (10)
	Master’s Degree	2 (10)	0 (0)	2 (7)
	Doctoral Degree	0 (0)	1 (10)	1 (3)
Time since diagnosis	< 1 year	4 (20)	3 (30)	7 (23)
	1–5 years	14 (70)	6 (60)	20 (67)
	> 5 years	2 (10)	1 (10)	3 (10)
Primary site	Oesophagus	2 (10)	1 (10)	3 (10)
	Stomach	0 (0)	0 (0)	0 (0)
	Hepatobiliary or pancreatic	6 (30)	3 (30)	9 (30)
	Large Intestine	11 (55)	6 (60)	17 (57)
	Other	1 (5)	0 (0)	1 (3)
Currently on treatment	Yes	19 (95)	9 (90)	28 (93)
Lines of treatment	One	6 (30)	3 (30)	9 (30)
	Two	6 (30)	4 (40)	10 (33)
	Three or more	8 (40)	3 (30)	11 (37)
Caring responsibilities	No	13 (65)	0 (0)	13 (43)
	Yes	7 (35)	10 (100)	17 (57)
Relationship to person	Child	0	2 (20)	2 (7)
	Parent	3 (15)	1 (10)	4 (13)
	Spouse/partner	3 (15)	7 (70)	10 (33)
	Other	1 (5)	0 (0)	1 (3)
Zarit Burden Interview (ZBI-12)^c^	Median, interquartile range		18 (10)	
	Mild burden		1 (10)	
	Mild-moderate burden		5 (50)	
	High burden		4 (40)	

^a^Modified Monash model describes an area based on geographical remoteness and town size. Areas classified MM2 to MM7 are considered regional, rural, or remote. People living in these areas can experience greater difficulty in accessing medical care

^b^Socio-economic indexes for areas (SEIFA) quintile ranks areas according to their relative socio-economic advantage, with higher scores indicating advantage and lower scores indicating relative disadvantage

^c^ZBI-12 scores: 0–10 indicates mild burden, 10–20 indicates mild to moderate burden, and > 20 indicates high burden

## Results

In total, 43 participants (26 patients; 17 caregivers) were contacted for interview, of whom 30 agreed to participate (20 patients; 10 caregivers). Demographic characteristics are summarised in Table 2. Thematic analysis revealed five main themes across groups: (1) treatment as work, (2) opportunity costs of receiving care, (3) treatment time as an investment, (4) time in treatment decision-making, and (5) tools for managing treatment time.

**Table 2:** Additional quotations illustrating major themes

Theme	Illustrative quotations
Treatment as “work”	“Even on these break periods I've had over the years I'm still back and forth to the hospital for blood tests, check-ups, scans all sorts. So I know that is admittedly easier than doing chemo every two weeks, but it's still something to do, something to think about” (P17-M) “It’s sort of like…. [patient] is the Sun, and I can be any of the other planets depending on what circumstances it might be. I might be really close, or I could be Pluto, over here doing the shopping and stuff and then have to slot all the way back” (C12-M) “I have to drive to (remote town) at 05:00 AM-05:30 AM Monday to be there for 05:40 AM… then we all come down in a little mini bus….(I spend) Monday, Tuesday doing the treatment… then a couple of days with that bottle on too. Thursday 3:00–3:30PM or something theyre taking the bottle off me… It’ll be something like 9:00PM or something by the time I get home….. and I’m thinking, every two weeks, jeez – is it worth it?” (P32-R) “One thing I find quite amazing is that they leave, say (home town) hospital and they go to (referral hospital), and then we've got to start all the way back at the start again: ‘So, what what brings you here to the ED’ and you go through the whole rigmarole of what her condition is and then they haven't got the charted meds there….” (C24-R)
Opportunity-costs of receiving care	“It takes me a week to get over it…. And by the time I feel good, you've got to go and get another one you know?” (P35-R) “I whinge about work, I whinge about having to go…. But I guess it’s a sign of normality, it’s sort of the thing that has been most taken away I suppose… and I guess probably I’m a little worried that I might not be able to get back” (P10-M) “Sometimes I just stop the car and… I just have this big cry to let things out… I don’t like (patient) to see me cry or upset, and so I hide a lot of feelings and I try to be very, very strong for him. And I don’t want him to worry…. I’ll worry about me later on” (C38-R) “I'm along here for the ride…. But I don't know where the sat-nav is taking me” (C12-M)
Treatment time as an investment	“I live with such… I'm so grateful. That is the overwhelming feeling. Does it take time? Does it consume my life from time to time? Yes, of course it does. Yeah, but the pay off is that I'm still here, you know what I mean?” (P6-M) “He's got to see another the granddaughter born. First year when he was diagnosed and he's got to see her, she's turned one where some people aren't that lucky. So he's lucky he got and that's the last grandchild we're going to get so (laughs)” (C30-R)
Time in treatment decision-making	“You know, somebody gives you a death sentence. You'll try anything”. (P35-R) I see now that it has just bought time…. I wish I had realised we really were buying time…. you know, he wanted to be positive and be maybe in a bit of denial (he) got all: ‘you know what I could beat this,’ even though we knew we were buying time. I wish I really, really had taken that on board…. I would push harder to get (our) stuff sorted”. (C15-M) “We should be open about all the benefits and all of the negatives – which include time – so that people can kind of make their own sensible judgement about….whether this is what they want to do…I think for the majority of people, it's probably really relevant to be a bit more thoughtful about; This is not just about days… it's really talking about quality of life”. (P44-M)
Tools for managing treatment time	“(through tears) when he had crappy news to deliver, he came and did that on his own, rather than with an entourage… And he made the time to sit down, and you know, I didn’t feel the conversation was rushed. I thought he was fantastic”. (P44-M) “(Remote, video accessed chemotherapy) saves going to (large centre)…. If I can have it all done in (hometown), that’s fabulous”. (P23-R)

### Treatment as work

Both patients and caregivers described cancer treatment as the central focus of their lives. Time itself was conceptualised in relation to chemotherapy cycles: “my life just revolves around a three-week cycle” (P37-R). Analogous to ‘work’, participants expressed a sense of resignation to treatment, with patients perceiving that they had “no choice” but to embark upon treatment to survive. Similarly, caregivers described “working their lives” around the emotional and practical needs of care recipients: “all my time is spent running the household… making sure (patient) is okay” (C38-R). Psychological reactions to illness intersected with perceived burdens from cancer care, whilst some accepted a “new reality” others fought vigorously to dichotomise their “cancer world” from their “normal world”.

### Burdens related to healthcare time

Chronologic time spent receiving healthcare was related to perceived burdens of outpatient care. Frequent, uncoordinated appointments—especially infusional treatments—were associated with a higher burden. Oral, self-administered treatments were often preferred by patients; however, caregivers found coordinating these treatments more onerous. Whilst patients appreciated treatment breaks, treatment-related healthcare tasks (e.g. port flushes) were intrusive: “it’s still something to do, something to think about” (C17-M). Ancillary tasks such as commuting and waiting for appointments were a major source of frustration, particularly for regional and remote participants: “You spend your whole life travelling…. We lose three days a week sort of thing” (P35-R).

Chronologic time burdens were amplified by experiential aspects of healthcare contact. Participants noted that healthcare contact was often most intensive near the time of diagnosis. Communication inefficiencies, late scheduling changes, and delayed receipt of imaging results contributed to perceived burdens. Some participants conveyed that routine appointments lacked the length and focus to appropriately address their complex care needs: “the whole oncology system is very narrow and quite broken” (P44-M). Unplanned, emergent, or inpatient care was described by some carers as a “nightmare” (C24-R), due to distress arising from patient-system conflicts such as perceived medication errors, long wait times, frequently repeating information, or having to advocate for appropriate care, yet they felt compelled to seek care for unwell relatives: “even though he’s there, I’ve still got to look after him…. And do all the other things I need to do at home!” (C15-M).

### Opportunity costs of receiving care

The burdens from healthcare time overlapped with perceptions of the opportunity costs of healthcare time. This was a topic participants were initially reticent to discuss, as the prevailing view was time spent receiving treatment was unavoidable. However, treatment days were frequently described as a “write off” (C16-M) with flow-on effects to competing commitments such as work, study, family, and travel ambitions:

Everything revolves around the cancer treatment. Because of your time that you've got to be here for it. You can't really go anywhere. You can't do anything. You take a while to recover. It all revolves around the chemo (P5-M)

There was heterogeneity amongst how strongly participants identified with this theme. Some participants described subtle, cumulative erosions of their pre-treatment identities. Others vocalised explicit concerns about the impact of healthcare contact time on their ability to pursue important activities:

It's taking up my life [becoming irritated] ….. you got limited life left and you spend most of it going back and forth to hospitals …. (it’s) quite frustrating because - I've got these things in my head I've got to do, (P35-R)

Treatment toxicities profoundly affected the quality of ‘home time’ for patients. The cumulative intensity of side effects was demoralising for patients: “there is an element of death by a thousand cuts” (P46-M). Caregivers reported a sense of being ‘on call’ for treatment and disease-related side effects: “you’re anxious through the night in case you hear anything, you know, just you've always got your door open just in case there's an issue” (C16-M). A crippling sense of uncertainty further tainted enjoyment of ‘home time’ for some patients and caregivers, the latter who expressed high degrees of emotional distress due to competing requirements of healthcare, grief reactions to illness, and the ongoing need to provide for loved ones:

(through tears) When you got so much to deal with It's hard to, it's hard to have the energy to work through it…. I `felt like I was the most capable person in the past, but I feel so disempowered with all the challenges. (C15-M)

### Treatment time as an investment

Despite the burdens of treatment time, healthcare time was judged as an investment in improving length and/or quality of life. Few participants expressed treatment regret, and many reflected that the ‘ends justified the means’:

for me it's more about what's going to do the job. If there was something five times more onerous that was going to be, you know, effective in getting rid of it, I'd sign up. Absolutely. (P44-M)

Some participants did not perceive treatment time as being burdensome: “I don't find it a burden. And I know I'm here for a reason…. I'm not here wasting my time” (P1-M). This view was more common in those with fewer treatment-related complications and patients with lower GI cancers. Psychological benefits of receiving treatment, such as providing hope, camaraderie, and assuaging existential anxiety, were mentioned. There was a broad sense of gratitude for oncology providers and the opportunity to receive care. Most perceived that the term ‘time toxicity’ was unhelpfully value laden and did not necessarily reflect their experience with attending healthcare.

### Time in treatment decision-making

Potential survival benefits, side effects, effect on quality of life, and clinician recommendation were generally held as being ‘drivers’ of treatment decision-making. Overall, treatment time and logistical considerations were considered ‘modifiers’ of treatment decision-making and were mentioned more frequently in patient interviews, especially at extremes of age:

I asked her how long… How long will I have to carry on like this? (P11-M)

Preferences about information delivery were discussed at length, with participants valuing information about treatment scheduling and duration of contact time per treatment episode and treatment course. Whilst scheduling was discussed comprehensively by clinicians, many participants expressed surprise about the length of intravenous infusions and duration of planned treatment: “I feel like they’ve got a plan but they don’t tell you the plan” (P10-M). Barriers to communication about treatment time included a lack of insight into prognosis, treatment intent, and initial ‘information overload’:

It's such a shock to the system….. It's so much information overload that I think you can't absorb it all…. So it doesn't really matter at that stage. But later on…. (P5-M).

### Tools for managing treatment time

Participants described a number of strategies for minimising the burden of healthcare time, including using idle time for self-care, leisure activities, or household tasks. Psychological coping included task orientation, compartmentalisation, denial, or minimising the impact of missed opportunities due to treatment. Many participants found continuing to work or study throughout treatment helped preserve their pre-treatment identity. Ultimately, relinquishing control and recalibrating future aspirations formed a critical part of accepting changed realities:

Why hate something that you cannot control? As soon as you give something like that control, you end up spiralling out of control and then you're not supportive. (C12-M)

Participants provided feedback on ways to reduce treatment time burdens, including upfront communication about planned healthcare time burdens or delays, coordinating appointments to occur on 1 day, or access to at-home services. Accessing home-care did not negate the sense of ‘work’ associated with treatment activities: “you’ve still got to be at home and wait for that person” (P17-M). Access to caregiving support, governmental financial assistance, and nurse coordinators was perceived as enormously beneficial to caregivers. Regional participants favoured telehealth and remote video–accessed chemotherapy to reduce time and logistical burdens. Suggestions for improving hospital layout, such as signposting and providing mobility aids at entrances, were also mentioned.

## Discussion

There is a growing body of quantitative literature examining healthcare contact time associated with receiving cancer treatment. This has been provocatively termed the ‘time toxicity’ of cancer treatment, although patient and caregiver perspectives on healthcare contact time are still being established. This study contributes the first qualitative analysis of the impact of healthcare time on patients and caregivers outside of North America, including the perspectives of participants in metropolitan Sydney and a regional centre serving a geographical area larger than Great Britain. Our aim was to capture a broad understanding of patient and caregiver perceptions of healthcare treatment time and understand how these may influence decision-making about palliative systemic cancer therapies. Our results suggest that time ‘toxicity’ is a subjective expression of the time impact of treatment, filtered through perceptions of treatment benefit, downsides, psychological reactions to illness, and sociocultural attitudes toward treatment. This study provides further nuance to the notion that all healthcare contact is ‘toxic time’, supporting the ongoing use of neutral language in academic discourse whilst elaborating the sources and consequences of healthcare contact time on patients and caregivers with advanced GI cancer.

The current findings reinforce previous research substantiating the quantitative time costs and perceived burdens of healthcare contact. Our cohort described receiving treatment as analogous to ‘work’, with the number, frequency, and length of appointments as well as total time on treatment relating to perceived burden [5, 10]. Patients described spending multiple days per treatment cycle in contact with healthcare, reflecting literature which shows patients with advanced GI cancer may spend one in 4 days in healthcare contact [2, 12, 21, 25]. Time burdens were shared by caregivers, who accompanied patients to appointments, acted as advocates, and coordinated healthcare visits. Reflecting the complexity and multidisciplinary nature of modern cancer care, participants reflected that the frequency of healthcare contact was often highest at diagnosis and reduced once established on treatment, which supports literature describing a ‘U’ shaped trajectory of healthcare contact during an advanced cancer diagnosis [11, 20, 25]. Whilst participants identified value in clinic appointments and treatment time, non-therapeutic but essential logistical tasks such as commuting, parking, and waiting for healthcare were particularly burdensome [5]. Half our participants were from a regional area, with 20% living remote [1] from specialist medical services. These participants described significantly higher burden from travel time, reinforcing the impact of travel burdens on engagement with cancer care in regional Australia [18, 26–28]. Telehealth and remote video–accessed chemotherapy helped to alleviate this burden.

An important finding of our study was the sense of ‘work’ associated with receiving treatment extended far beyond direct healthcare time costs. As in other studies, we found cancer care commonly commanded a central role in participants’ lives, with opportunity costs for participants’ engagement with work, home, and personal lives [14]. Unlike other work focusing on time burdens, we found the quality of home time was extensively affected by the morbidity of GI malignancies and their treatment [5, 14]. Consistent with previous research, caregivers were also affected by the effects of treatment and disease-related complications and often felt consumed by the tasks of running a household, providing direct patient care, and coordinating treatment activities. Interestingly, participants’ perceptions of treatment burden were only partially mitigated by providing care in the home; many lamented having to ‘work their lives’ around cancer care and sought to avoid ‘reminders’ of their illness. This suggests that the time burdens of care extend beyond what is easily measurable (healthcare contact days) and encompass further indirect time costs, compounding the opportunity costs of receiving treatment for patients and their caregivers.

Affronted by life-threatening illness, participants felt that their treatment choices were constrained by the need for treatment to survive. It followed that participants reported prioritising survival time, side effects, and quality of life over healthcare time and logistical considerations when making treatment decisions. However, many identified an understanding of healthcare contact time requirements as being important for planning their lives. Whilst a growing body of literature reports healthcare time associated with treatment, further quantification of healthcare time in real-world and trial populations is warranted. Ideally, measures of healthcare contact should form part of mandatory reporting in clinical trials, as these may inform assessments of treatment value to individual patients. Whilst this study illuminates stakeholder perceptions on healthcare time, quantitative weighting of decision-making factors was not possible; this will be evaluated in a subsequent discrete-choice experiment.

Participants in our study perceived healthcare time could be a meaningful time investment, reporting that treatment had allowed them to achieve important milestones in the context of a life-limiting illness. Despite the potential burdens of healthcare, many had overwhelmingly positive experiences with care providers, and some were reluctant to reflect on negative aspects of healthcare time. When challenged to reflect on the term ‘time toxicity’, most found it did not reflect their healthcare experience and was jargonistic and unhelpfully value laden. This nuance provides additional context to time toxicity literature, which initially framed healthcare contact in the negative [8], but has moved toward more neutral descriptors such as ‘contact days’ [11]. Clinicians should be cognisant of when communicating with patients and caregivers about healthcare time associated with treatment.

Encouragingly, the sources of treatment time burden reported by participants in our study are potentially modifiable. First, patients and caregivers valued clear and timely communication about wait times, delays, and appointment changes. Efficient communication between clinicians, including optimal use of electrical medical records by clinicians, may reduce the burden of information sharing on patients and caregivers. The use of electronic patient portals may assist some patients and caregivers in viewing and managing upcoming appointments, saving additional time coordinating appointments. Second, patient-centric organisational layouts may reduce the time and effort required for routine tasks, for example, running multidisciplinary clinics where patients can see multiple providers in one place, having mobility aids at entrances, minimising the distance between interrelated services (e.g. oncology departments and medical imaging), and clear signposting. Third, care delivery can be optimised by using oral or subcutaneous treatments, telehealth technologies, and home-based services and improving access to patient navigators, nursing support, and psychosocial care for patients and caregivers facing high levels of carer distress. Reducing excessive noise, light, and medical interventions during rest times may ameliorate the burdens of inpatient healthcare time. In addition to healthcare system–based issues, the significant impact of side effects underlines the importance of inclusive clinical trial design, unbiased reporting of toxicity, and innovation in supportive care as well as anti-cancer drug therapies.

Our study has several limitations. Although purposive sampling was used, there was a paucity of younger patients and caregivers. Only participants from public hospitals within one Australian state, who had received cancer treatment, were interviewed, and views expressed may not be generalisable to those who chose not to have treatment or other sociocultural contexts, cancer types, or healthcare systems. Further, we did not capture the perspectives of First Nations and culturally and linguistically diverse populations.

This study provides a foundational understanding of time toxicity from the perspective of Australian patients and their caregivers. In highlighting stakeholder viewpoints, the sources and consequences of time toxicity, this research supports efforts to identify, assess, and address modifiable sources of time burdens in cancer care.

## Supplementary Information

Below is the link to the electronic supplementary material.

Supplementary file1 (DOCX 39 KB)

## Data Availability

The data underlying this article cannot be shared without compromising the privacy of individuals who participated in the study. The qualitative nature of the interviews and experiences of clinicians are personal even if identifying information is removed from individual transcripts, it may still breach confidentiality.
