# Supplementary material for: “The Cancer is My Life”: patient and caregiver perceptions of the time toxicity of palliative systemic cancer treatments for advanced gastrointestinal cancers
Source: Support Care Cancer. 2025 Jun 10;33(7):564. doi: 10.1007/s00520-025-09621-4 (PMC12152011; doi:10.1007/s00520-025-09621-4)
Supplement: Supplementary file 1 — Supplementary file1 (DOCX 39 KB) [file 520_2025_9621_MOESM1_ESM.docx]

**Supplement 1: COREQ Checklist**

| Domain 1: Research Team and Reflexivity  Personal Characteristics | | | |
| --- | --- | --- | --- |
| 1 | Interview | Which author(s) conducted the interviews or focus group? | One researcher carried out interviews (SS) |
| 2 | Credentials | What were the researcher’s credentials? | SS is a PhD candidate, holds an MBBS (Hons) and is a Fellow of the Royal Australasian College of Physicians |
| 3 | Occupation | What was their occupation at the time of the study? | SS is a medical oncologist and PhD Candidate employed as a research fellow. |
| 4 | Gender | Was the researcher male or female? | SS is male. |
| 5 | Experience and training | What experience or training did the researcher have? | SS was instructed on interview conduct JS, who is an experienced psycho-oncology researcher. This included practical training. JS provided additional indirect feedback via review of interview transcripts. |
|  | Relationship with participants | | |
| 6 | Relationship established | Was a relationship established prior to study commencement? | SS had no pre-existing or ongoing relationship with participants. |
| 7 | Participant knowledge of the interviewer | What did the participants know about the researcher? | Participants knew where the researchers worked and purpose of the research. It was disclosed that this research would contribute to SS partially satisfying the requirements of a PhD |
| 8 | Interviewer characteristics | What characteristics were reported about the interviewer/facilitator? | The main researchers had an interest in the research, and this was declared in the PIS |
|  | Domain 2: Study Design | | |
|  | Theoretical framework | | |
| 9 | Methodological orientation and theory | What methodological orientation was stated to underpin the study? | Grounded theory was used. An inductive approach to analysis was taken, using thematic analysis, tied to a framework approach. A team-based, reflexive approach was used to coding, using the constant comparison method. |
|  | Participant selection | | |
| 10 | Sampling | How were participants selected? | A purposive sampling strategy was used as described in the manuscript. The researchers contacted the treating oncologists to identify potentially suitable patients and caregivers for interview. |
| 11 | Method of approach | How were participants approached? | Oncologists were initially informed of the study via departmental presentation and email invitation. They were encouraged to approach potentially eligible participants during routine clinical encounters. After verbal consent was provided, participants were contacted by a member of the research team. |
| 12 | Sample Size | How many participants were in the study? | Thirty participants took part in semi-structured interviews (20 patients and 10 caregivers), or until data saturation was confirmed by the research team. |
| 13 | Non-participation | How many people refused to participate or dropped out? Reasons | Patients who were initially approached who subsequently refused to participate were recorded in the study database. 20/23 patients and 10/11 caregivers who initially approached participated in interviews. Refusal reasons were not required. |
|  | Setting | | |
| 14 | Setting of data collection | Where was the data collection? | Face-to-face interviews were conducted in a private clinical setting; online-interviews were conducted using Microsoft Teams |
| 15 | Presence of non-participants | Was anyone else present besides the participants and researchers? | Only the researchers and participants were present |
| 16 | Description of sample | What are the important characteristics of the sample? | Important demographic information is summarised in the manuscript |
|  | Data collection | | |
| 17 | Interview guide | Were questions, prompts, guides provided by the authors? Was it pilot tested? | Semi-structured interview guides were developed by the researchers in consultation with a patient advocate and used during interviews. These were pilot tested in the initial interviews. |
| 18 | Repeat interviews | Were repeat interviews carried out? | No repeat interviews were required |
| 19 | Audio/visual recording | Did the research use audio or visual recording to collect the data? | Interviews were audio recorded using Microsoft Teams |
| 20 | Field notes | Were field notes made during and/or after the interview? | No field notes were routinely collected. |
| 21 | Duration | What was the duration of the interviews? | Interviews ranged from 25 to 60 minutes. |
| 22 | Data saturation | Was data saturation discussed? | Transcripts were reviewed and coded using the constant-comparative method. A teams based reflexive approach was used to continually assess for data saturation. |
| 23 | Transcripts returned | Were transcripts returned to participants for comment and/or correction? | To ensure fidelity to the participants’ original impressions, transcripts were not returned for review. |
|  | Data analysis | | |
| 24 | Number of data coders | How many data coders coded the data? | Two researchers (SS and EE) coded transcripts independently, as described in the manuscript. Coding schemas were refined iteratively in consultation with JS and JV. |
| 25 | Description of the coding tree | Did authors provide a description of the coding tree? | A coding tree is provided in Supplement 3 |
| 26 | Derivation of themes | Were themes identified in advanced or derived from the data? | Themes were derived from the data. |
| 27 | Software | What software, if applicable, was used to manage the data? | NVivo 14 for Mac, licensed to the University of Sydney. |
| 28 | Participant checking? | Did participants provide feedback on the findings? | No |
|  | Reporting | | |
| 29 | Quotations presented | Were participant quotations present to illustrate the themes/findings? Was each quotation identified? | Quotations have been presented throughout the manuscript and further quotations used to illustrate themes in the codebook (Supplement 4) |
| 30 | Data and findings consistent | Was there consistency between the data presented and the findings? | We endeavoured to report the study findings in a clear consistent manner to accurately reflect the data that have been collected. |
| 31 | Clarity of major themes | Were major themes clearly presented in the findings? | Yes, major themes are clearly presented in the manuscript. |
| 32 | Clarity of minor themes | Is there a description of diverse cases or discussion of minor themes? | Yes, all data relating to the development of coding is presented in the manuscript and Codebook (Supplement 4). |

**Supplement 2: Interview Guide**

| Patients |
| --- |
| - [Referring to demographic and treatment information] Tell me about what impact the diagnosis has had on your life. - Can you explain what an average day is like when you are going to the hospital for treatment?   - Probes: blood tests; clinic appointments; treatment administration; scans; commuting; form filling - Have there been times during your treatment when you have experienced debilitating side-effects?   - Probes: impact on day-to-day life, requirement for health care attendance - Are there elements of receiving treatment that often take longer than expected?   - Probes: check-in; clinic; pre-med delays; blood test clerical errors; treatment delays for toxicity - What aspects of cancer care cause you the most frustration and distress? - When you are not doing things for your health, how do you like to spend your time? - What impact does the time you spend getting healthcare have on the rest of your life? - Do you feel that you miss out on things because you are having to do things related to your medical treatment?   - Probes: family time; work; leisure; travel; important events - Think back to when you started your most recent cancer treatment. Can you walk me through what sorts of things were important to you when considering your new treatment?   - Probes: efficacy; side-effects; logistics and convenience; novelty; financial cost; impact on daily activities, personal, family, work, or study commitments; emotional or cognitive effects - Back when you started treatment, what were your expectations about how much time would be involved in receiving treatment?   - Probes: were time considerations discussed?; has it turned out to be in line with your expectations? - Would you appreciate knowing about time-in-treatment in the future? - If you knew what you now know about the time involved in your cancer treatments, would you make the same choices again? - Overall, how do you feel about the time needed for you to coordinate and receive treatment? - Sometimes we use the word ‘toxicity’ to talk about the side effects of a treatment. The term 'time toxicity' has been used to describe the impact of the time associated with receiving cancer treatment. What are your reflections on this term?   - Probes: would you propose an alternative term? |
| Caregivers |
| - [Referring to demographic and treatment information] In your role as a care giver or support person, what type of activities do you help with? - Do you accompany [patient] when they have healthcare appointments or treatment? - Can you explain what an average day is like when [patient] goes to the hospital for treatment?   - Probes: blood tests; clinic appointments; treatment administration; scans; commuting; form filling - Have there been times during your treatment when you have experienced debilitating side-effects?   - Probes: impact on day-to-day life; requirement for health care attendence - Are there elements of [patient’s] treatment that often take longer than expected?   - Probes: check-in; clinic; pre-med delays; blood test clerical errors; treatment delays for toxicity - What aspects of cancer care cause you the most frustration and distress? - What other responsibilities do you have apart from your role as carer?   - Probes: work; parenting; other carer responsibilities; community roles - How has [patient’s] illness and treatment impacted on your day-to-day life? - Are there things that you feel you miss out on things because of attending appointments or treatments with [patient]? - Think back to when [patient] started their most recent cancer treatment. What was your level of involvement with that decision? Probes: were you present for the discussion? What kind of conversation did you have afterwards? What was that like for you? - Thinking about that decision, what kind of characteristics of the treatment were important to you as the support person for [patient]?   - Probes: efficacy; side-effects; logistics and convenience; novelty; financial cost; impact on daily activities, personal, family, work, or study commitments; emotional or cognitive effects - Do you remember the doctor mentioning how much time [patient] would need to spend at the hospital or doing tests? - Considering everything that is involved, how do you feel about the time associated with [patient’s] healthcare? - If you knew what you now know about the time involved in [patient]’s cancer treatments, would you make the same choices again? - Overall, how do you feel about the time needed for you to coordinate and receive treatment? - Sometimes we use the word ‘toxicity’ to talk about the side effects of a treatment. The term 'time toxicity' has been used to describe the impact of the time associated with receiving cancer treatment. What are your reflections on this term?   - Probes: would you propose an alternative term? |

**Supplement 3: Framework Analysis**

| Transcription | In this study, transcription was carried out by two authors (SS and EE). Transcription was initially performed automatically, using the online transcription service TRINT ([www.trint.com](http://www.trint.com)). SS and EE then examined transcripts for accuracy. We were primarily interested in the content of responses, thus, only long pauses, interruptions, non-verbal communication (laughter, crying) were noted within the text. Transcripts were checked for errors by peer review and clarification in regular research meetings. |
| --- | --- |
| Familiarisation | Four members of the research team reviewed initial transcripts (SS, EE, JV, JS) to refine the question guide and provide feedback on interview style. SS and EE read and re-read all transcripts and listened to audio-recordings to become familiar with the whole dataset. Initial impressions were recorded using the annotation function in NVivo prior to coding. |
| Coding | Two members of the research team (SS, Medical Oncologist and PhD Candidate, and EE, Pharmacist and Medical Student) coded the same three transcripts in each cohort before meeting to discuss emergent codes. Codes consisted of short phrases to whole paragraphs describing a particular phenomenon or idea. We used a constant comparative method to ensure consistency of coding, with regular review by senior authors (JS and JV). |
| Developing a working analytical framework | After SS and EE had each coded the same three transcripts in each cohort, we met to discuss the codes we had used to describe the data. We discussed emergent ideas and constructed an initial coding framework in consultation with JS and JV. Disagreements about coding labels were discussed and consolidated, with input from a senior author if needed. A brief description was included in the codebook for consistency. Using this framework, a further three transcripts each were coded, before further meetings to revise and refine the framework. This process was repeated until no new themes were generated and the final thematic framework was agreed. |
| Applying the analytical framework | We applied the final analytical framework to each transcript by importing transcripts in QSR NVivo version 14 for Mac and attaching appropriate codes from the analytical framework. All authors participated in organising codes into categories reflecting prominent themes within the data set. |
| Charting data into the framework matrix | A matrix was presented for each theme by abstracting, summarising and charting data for each case and each code within that theme. |
| Interpreting the data | Thematic analysis was carried out on the data set by reviewing matrices and making connections within and between codes and participants. This process allowed for explorations on how patients and caregivers within our dataset experienced the perceived and experienced the phenomenon of ‘time toxicity’. |

Supplement 4: Additional Quotations

| Theme 1: Treatment as ‘work’ | |
| --- | --- |
| Subtheme | Node*, Illustrative Quote |
| Organising life around treatment | “You know I worked my life around. You know, every fortnight, I just worked my life around my chemo treatment, and I still do.” (P1-M)  “My life just revolves around a three week cycle…. That’s it. I’ve got three weeks to live” (P37-R)  “The cancer is my life at the moment. There's no way of avoiding it. But yes, there is a level that you want to keep separate. You know, you don't need everything to be cancer-related and what not. You do want, just: normal life. You just want to keep doing other things. So yeah, it’s definitely… It's got to a point of acceptance almost… You know, cancer is what life is. But, you know, there's also life outside of cancer. We want to keep that.” (P17-M)  “I don't have any hobbies or going to see other people or anything like that. It's it's mainly because all my time is spent running the household, doing housework. Making sure (patient) is Okay. he's priority. Everything starts with him.” (C38-R) |
| Sources of burden | Node: Chronologic time burdens  ''It's not easy to come to hospital every day, and sometimes you have to come to hospital two or three times a week. There was a time where I had to come three days in a row on Tuesday, Wednesday, Thursday, but you have to come.” (P3-M)  Node: Commuting  “(We) stay (near the hospital).... Before the night of the chemo so that we didn't have as far to travel in the morning and then, leave from there in the morning and head in to (hospital) for the treatment... I guess (it’s) a bit like a three day process.” (C24-R)  Node: Coordinating care  “I just get sick…. Sick of it… trying to go and get stuff organized with the chemist, you know?” (P31-M)  Node: Psychological impact of treatment  “I come in, and most times I do have tears. So it's a reality, that whole reality of a walking hospital and you walk past the kiosk and I'll have tears…. you kind of know you got to do it and it's… but it hits you.” (P14-M)  Node: Self-administered treatments  “You know, the tablet – I have to, go to a chemist to buy it. Every time. The injection… We have to just go to the hospital and the chemo nurse does everything…. But the tablet, it’s more things I have to do: I have to make a phone call and check the blood test. But it’s less time at the hospital.” (C13-M)  Node: System versus patient priorities  “Radiology is chronically running late… (sometimes) when I’m sitting down waiting, they phone and they say: ‘where are you?’.” (C12-M)  “Sometimes it's hard when you've got two or three doctors on the same day. That makes it a bit hard. You could have an appointment, say, nine in the morning and one, two, three in the afternoon. And it’s the in between. It's not easy to go home. So you're stuck here all day, and having to deal with that.” (P3-M)  Node: Waiting for results (scanxiety)  “It’s just the CT Scans… They are my absolute dread…. I hate waiting for the results because it impacts my life. And that’s when I live with cancer outside of here. And that means I don’t sleep well, I don’t feel well, that affects my well-being and the last one that I had about a month ago…. It took me two weeks to get the report!... What doctors have to understand is…. the ripple effect on my life is huge!” (P6-M) |
| Theme 2: Opportunity Costs of Care | |
|  | Node, Illustrative Quote |
|  | Node: Effect of competing priorities on perception of time burdens  “I was under the pump then… I'd have to go in and do my bloods before I went to (work)…. And when you get there early there's a lot of people there…. I would have to wait and I'd be quite worked up about that because I'd have my number, and I'd be sitting there waiting because I knew I had an appointment, you know, at whatever time.” (P6-M)  “I'm obviously retired, so the time's not an issue. The issue is, one of my daughters generally has got to take a day off work to run me around” (P43-R)  Node: Effect of physical side-effects on home time  “I've found since (treatment) my energy levels just totally plummeted, like to get up in the morning, it could take anywhere, anywhere from an hour to two hours just to, like, showered and dressed.” (P23-R)  “Usually we'll just go and lie down and then your anxious through the night in case you're hearing things, you know, just you've always got your door open just in case there's an issue…. So… I do work around and about that; (I) basically block those three days at as time that anything could change.” (C16-M)  Node: Impact on home and family life  “As a parent it's been quite difficult. I haven't been able to fulfil home roles like: I was always very active with the kids and with their lives and.... I do struggle to walk the 100m or 50m from my house up to the shed…. and look, what I would love to do! Also sleeping a lot and resting.” (P14-M)  Node: Experience of time whilst on treatment  “When you got this crap, the time just flies. It's just. I dunno whether it's just what's happening to everybody or whether it's just me. You know, the last two years have been a blur…. It's just lost. I just lost a year there.” (P35-R)  Node: Impact on social life  “I don’t really go out to see friends anymore, because I think I’ll get more stressed, like… Keep checking on her.... So (I) just stay home.” (C13-M)  Node: Impact on travel ambitions  “We’re not living the life that we were meant to do with each other… (becoming melancholic) we can’t do the things that we had planned on doing…. I just wish this late in life – that I met this amazing man, that we were going to do the things, you know, have the honeymoon, and do the things that we had planned. But (sighs) it’s just not meant to be….” (C38-R)  Node: Impact on work and study  “So right in the middle of a kind of a crisis, basically. I mean, I have to start over again with a career. And it just it feels like a really tricky place to be because, you want to be up front when you do have interviews. Look,I can work full-time or part-time, but I might need every Wednesday off.” (C15-M)  “I used to think, ‘oh I’ll drop him and come back to do work’…. But I know now that day’s a write off because you’re always anxious, you’re always watching the phone, you’re always checking things are okay and then go down to pick him up. And it’s complex in Sydney because of traffic.” (C16-M)  Node: Effect of uncertainty  “When we got the diagnosis, all plans just ceased to exist. We just stopped everything…. The first year was just horrible, we were sort of…. You’re just expecting to die, more or less. And so, you just can’t really think about anything else. And you worry so much about what’s happening in your body…. You feel a slight ache in your belly…. And you just panic straight away: ‘oh my God, I’m done for now’.” (P37-R) |
| Theme 3: Treatment Time as an Investment | |
| Subtheme | Node, Illustrative Quote |
|  | Node: Treatment as a source of hope  “When my doctor says something like: ‘well we haven't really accessed quite a few treatments and you've been on this so long, so we've got choices here…’ That's what I need to hear! I need that! I need to hear about the choices, I need to hear a plan, and I need to know that there's hope. And that's, I think, for a lot of people, I should imagine, would be similar, because in my mind, it's this thing... when do you give up?” (P6-M)  Node: Necessity of healthcare time (the ‘ends justify the means’)  “I Guess you've got a choice. Do nothing, or try something. Let's try something to see if it improves..... If it extends my lifetime here, and gives me more time with the people I care about.” (P5-M)  “I live with such... I'm so grateful. That is the overwhelming feeling. Does it take time? Does it consume my life from time to time? Yes, of course it does. Yeah, but the pay off is that I'm still here, you know what I mean?” (P6-M)  Node: Relation of healthcare time ‘toxicity’ to treatment benefit  “If it was more effective, the time toxicity wouldn't be such an issue I don't think, you know?” (P35-R)  “When I had the radiation, it was every second day and it was like: driving here, driving home. And like, I don’t really want to go through that. You got to drive here, come through the traffic… If it’s going to be changing my life, or if it’s going to be important… I would still come.” (P4-M) |
| Theme 4: Treatment Time in Decision-Making | |
| Subtheme | Node, Illustrative Quote |
| Talking about treatment time impacts | Node: Barriers to effective communication about healthcare time  “He's more than willing to have treatment because he wants to get better. But he doesn't understand that he's not going to get better. But if it was me, yeah, I wouldn't have the treatment. I'd have the quality of life. Not the quantity.” (C49-M)  “As much as the oncologist talks to me…. I'm taking every third word in at this stage.” (P6-M)  Node: Expectations about healthcare time  “I had no concept. I actually hadn't even heard of oesophageal cancer before this really. So um no, I had no idea. I thought I could keep working through the chemo when I first started. When I first got it, I did go back to work. That probably wasn't good move. But, because I got so sick….. It does take a lot of time. But again, I don’t begrudge any of that. You just can’t live the way you used to.” (P14-M)  Node: Importance of discussing healthcare time  “In cancer, there's such a lot of unknowns. About your future, about the impact, about the time consumed. Everything. So if someone can put some of that and answer some of those questions.” (P6-M)  Node: Preferences about communicating healthcare time  “It’s been a conversation… Perhaps it would be nice if it was documented…. If we had something to refer back to it would be helpful.” (C15-M)  Node: Terminology of ‘Time Toxicity’  “I felt it could have been a more moderate terminology for the time involved: the time commitments? I don't know…. when I read that I went: ‘Oh, I just don't think that's a good description of how I would describe the time,’ because, to be honest, so for us up to this point, (patient) has had a really good response. The time involved is neither here nor there. It's been fantastic that he's had a response. So it's a positive time in a way.” (C16-M)  “I think it says a lot. It explains it pretty well, to be honest… I think that toxicity adds a level of negativity to it. But having to come in and do chemo for six hours, it’s not a positive thing to do. So I feel like, that ‘toxicity’…. It gives it a bit of… almost bluntness, feeling true like: this is what it is – it’s not a great thing, but we know that, whereas if it was another word, it might fluff over the fact of what (it is).” (P17-M) |
| Treatment Decision Making Factors | Node: Efficacy  “The efficacy of the treatment is number one, out and out.” (P44-M)  Node: Toxicities  “I was scared of all the after effects and the side effects of this treatment.” (P11-M)  Node: Recommendation from clinician  “Whatever the oncologist tell me to do, I will do” (P1-M)  Node: Effect on competing priorities  “(my main concern is)…. how is it going to impact my work, and how am I going to organise that around the treatment.” (P46-R) |
| Theme 5: Tools for Managing Treatment Burdens | |
| Subtheme | Node, Illustrative Quote |
| Healthcare factors | Effective Communication  “It’s really good. So much communication. I don’t get lost in the system… If I forget there’s an appointment, there’s always a follow up…. I always have the phone numbers to the people that I need… If I don’t get the person, then they call me back… Everybody in the MDT.” (C45-M)  Node: Financial Support  “I’ve got cancer assist [financial support]… So we’re pretty lucky… Accommodation (close to regional referral hospital) is basically free.” (P35-R)  Node: Psychosocial support  “I do talk to the cancer council support people, at oncology and the counsellor. So she's been very helpful…. Because it's very, very daunting when you first get diagnosed and you just don't know where to go, what to do or what's happening. And she was just wonderful. She helped us so much with services….. she was always there and you know send her an email. And yeah, it was very comforting to know and I could talk to her about how I felt.” (C38-R) |
| Individual behaviours | Node: Use of idle time  “(All ) the hours I spent getting FOLFOX and FOLFIRI. You know, initially in the early days, like six hours in the chair and stuff. I mean…. Once you get over the initial drama of it all, that time spent in the chair was quite relaxing. I mean, you can read, you could sleep.” (P6-M)  Node: Consolidating visits  “It’s better that (healthcare visits) are all on one day. So it’s one big day and then mum has time to recover.” (C7-M)  Node: Psychological strategies  “When I go for my training, my exercise, I forget that I have cancer. When I'm walking or riding, I just do that like self meditation or just being with the nature. I feel like nothing happened to me. It feels great.” (P2-M)  “In my mind: I come here, I do chemo, I have all these wonderful people that look after me and then I leave and I do my life. So my mind only allows me to deal with.... I guess is cancer coming in and out of it – in my mind, of course. Of course, cancer is part of life, but I never allow it to overtake it. When I'm here, it has to. When I leave, it stops. So the (5-Fluorouracil) bottle and walking away and going into my home - that is quite challenging for me because that challenges that thought process. Does that make sense? That's coming in to my life. So not that I - well I think if that's what I've got to do, that's what I'm going to do. Because that's it. I'll drive myself back and get the bottle off and off I go again. I did do that before, years ago, this particular regimen so, it's keeping me alive.” (P6-M) |
